# Cellular and Axonal Diversity in Molecular Layer Heterotopia of the Rat Cerebellar Vermis

**DOI:** 10.1155/2013/805467

**Published:** 2013-09-26

**Authors:** Sarah E. Van Dine, Elsaid Salem, Elizabeth George, Nga Yan Siu, Timothy Dotzler, Raddy L. Ramos

**Affiliations:** ^1^Department of Biomedical Sciences, New York Institute of Technology College of Osteopathic Medicine, Northern Boulevard, P.O. Box 8000, Old Westbury, NY 11568, USA; ^2^Department of Life Sciences, New York Institute of Technology, Old Westbury, NY 11568, USA

## Abstract

Molecular layer heterotopia of the cerebellar primary fissure are a characteristic of many rat strains and are hypothesized to result from defect of granule cells exiting the external granule cell layer during cerebellar development. However, the cellular and axonal constituents of these malformations remain poorly understood. In the present report, we use histochemistry and immunocytochemistry to identify neuronal, glial, and axonal classes in molecular layer heterotopia. In particular, we identify parvalbumin-expressing molecular layer interneurons in heterotopia as well as three glial cell types including Bergmann glia, Olig2-expressing oligodendrocytes, and Iba1-expressing microglia. In addition, we document the presence of myelinated, serotonergic, catecholaminergic, and cholinergic axons in heterotopia indicating possible spinal and brainstem afferent projections to heterotopic cells. These findings are relevant toward understanding the mechanisms of normal and abnormal cerebellar development.

## 1. Introduction

The lamina and folia of the cerebellum emerge from the precisely orchestrated proliferation and migration of neurons and the organized growth of neuronal elements including axons and dendrites [1–4]. In light of several devastating malformations of human cerebellar development affecting posture, balance, and motor learning [5–8], the molecular and genetic mechanisms of cerebellar lamination and foliation have been topics of intense investigation. Studies using rodent models have been extremely valuable in the understanding of human cerebellar development and the underlying mechanisms of cerebellar malformations. 

Several strains of rats exhibit spontaneous malformation of the primary fissure (PF) of the cerebellar vermis [9–14]. Malformations are characterized by collections of  heterotopic cells present in the molecular layer toward the base of the PF, suggestive of migration defect by granule cells exiting the external granule cell layer during early postnatal periods. Consistent with a model of neuronal migration defect as the cause of molecular layer heterotopia (MLH), Bergmann glia in heterotopia exhibit morphological abnormalities with radial fibers that fail to reach the pial surface [11] or with fibers that inappropriately cross into the adjacent molecular layer [13]. MLH are also characterized by abnormal lamination and dendritic organization of Purkinje cells [11–13].

Greater knowledge of the cellular and axonal anatomy of heterotopia of the PF has important implications toward our understanding of normal cerebellar development as well as cerebellar disorders with defective neuronal migration and altered lamination. In the present report, we further describe the cellular and axonal anatomy of MLH in the rat cerebellum using immunocytochemistry and histochemistry. In particular, we identify parvalbumin-expressing molecular layer interneurons in heterotopia as well as three glial cell types including Bergmann glia, Olig2-expressing oligodendrocytes, and Iba1-expressing microglia. In addition, we document the presence of myelinated, serotonergic, catecholaminergic, and cholinergic axons in MLH indicating possible spinal and brainstem afferent projections onto heterotopic cells. How heterotopia affect cerebellar function remains poorly understood; however, our data reveal that MLH contain diverse neurons, glia, and afferent axons.

## 2. Experimental Procedures

Sprague-Dawley rats were bred in our vivaria from founders obtained from Charles River Laboratories. Rats were housed in temperature- (20–22.5°C) and humidity- (20%) controlled facilities, with 12 hour light/dark cycles, in standard plastic cages with food and water available *ad libitum*. All measures were taken to minimize pain or discomfort in rats and experiments were carried out in accordance with the National Institutes of Health Guide for the Care and Use of Laboratory Animals (NIH Publications no. 80-23). All procedures were approved by the Institutional Animal Care and Use Committee at New York Institute of Technology.

Unless otherwise noted, adult rats greater than 6 months of age were deeply anesthetized and perfused with 0.1 M phosphate buffered saline (PBS) followed by a phosphate-buffered fixative containing 4% paraformaldehyde. Brains were removed from the calvarium and sectioned on a vibratome or cryostat. Brains sectioned on the vibratome were cut in the sagittal plane at a thickness of 60 *μ*m and collected in 0.1 M PBS. Prior to sectioning on the cryostat, brains were cryopreserved in a 30% sucrose solution in PBS. Brains were sectioned in the sagittal plane at a thickness of 45 *μ*m and collected in 0.1 M PBS. 

We followed histological methods identical to that described previously for mice [15, 16]. Immunocytochemistry was used to reveal the presence of different cellular and axonal phenotypes in heterotopia as previously described [15]. Briefly, free-floating sections were collected into different wells and washed with PBS (3 times). Sections were permeabilized and blocked in 5% normal goat serum (NGS) and 0.2% Triton X-100 for 1 h. Sections were incubated in primary antibodies with 0.1% Triton X-100 and 2.5% NGS in PBS at 4°C overnight. In the present study, the following primary antibodies were used: doublecortin (1 : 500, Santa Cruz), parvalbumin (1 : 1000), calretinin (1 : 1000; Millipore), serotonin transporter (1 : 1000; ImmunoStar), tyrosine hydroxylase (1 : 1000, Millipore), oligodendrocyte transcription factor 2 (1 : 1000, Millipore), nestin (1 : 200; Millipore), and ionized calcium-binding adapter protein 1 (1 : 1000, Millipore). Following incubation in primary antibodies (for use with brightfield microscopy), sections were rinsed several times with PBS and then incubated in biotinylated secondary antibodies raised in appropriate host species (1 : 200, Vector Laboratories) for 2 h at room temperature. Sections were rinsed 3 times with PBS and then incubated for 1 h in an avidin-horseradish peroxidase mixture. Sections were rinsed in PBS 3 times and then reacted with 0.05% diaminobenzidine in the presence of 0.0015% H_2_O_2_. For epifluorescence imaging, Alexa 488- or Alexa 568-conjugated secondary antibodies were used (1 : 200, Life Technologies). Fluorescent myelin staining was performed with FluoroMyelin (Life Technologies) according to the manufacture's protocol. Fluorescent counterstaining was performed with propidium iodide, DAPI, or Sytox green (Life Technologies). Sections from at least 3 brains were used for all antibodies tested.

Sections were mounted onto gelatin-coated slides and coverslipped. Digital photomicrographs were taken at varying magnification on an Olympus BX51 or Leica SP5 confocal microscope. Figures were prepared in Adobe Photoshop.

## 3. Results

**Figure 1:**
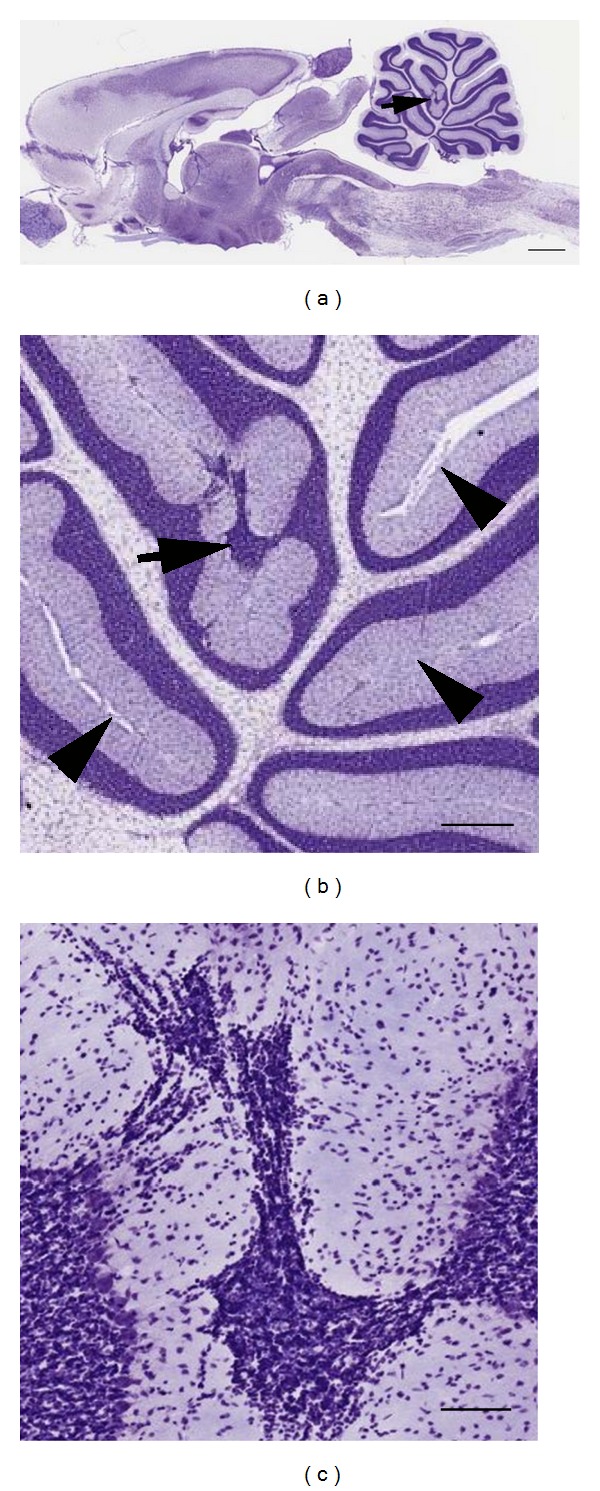
(a) Nissl-stained midsagittal section of the rat brain with a heterotopia of the primary fissure (arrow). ((b)-(c)) Higher magnification of heterotopia reveals a lack of pia at adjacent molecular layers compared to normal folia (arrowheads in (b)). Photomicrographs in (a)–(c) adapted were from (http://brainmaps.org/ajax-viewer.php?datid=107&sname=n12b). Scale bars in microns: (a) = 1472; (b) = 368; (c) = 92.

 MLH are found widely in Sprague-Dawley and Wistar rats from various vendors [12]. Therefore, while often overlooked by investigators, heterotopia are easy to identify. For example, a representative Nissl-stained midsagittal section taken from an online atlas of the rat brain is shown in Figure 1 (http://brainmaps.org/ajax-viewer.php?datid=107&sname=n12b). Along the primary fissure (arrow in Figures 1(a) and 1(b)), a heterotopic collection of Nissl-stained cells is evident in the molecular layers where normally a pial boundary would be evident (arrowheads in Figure 1(b)). Closer examination of Nissl-stained cells in heterotopia reveals the presence of cells with small somata indicating that the majority of cells in heterotopia are likely granule cells as previously suggested [13].

We recently described the cytoarchitecture [17] and axonal constituents [18] of MLH in the cerebellar vermis of mice using immunohistochemical techniques. Having identified several cellular and axonal markers that were useful in the description of heterotopia in mice, we used these same markers in the present study to describe additional cell types and axonal classes in MLH of the PF in rats. 

**Figure 2:**
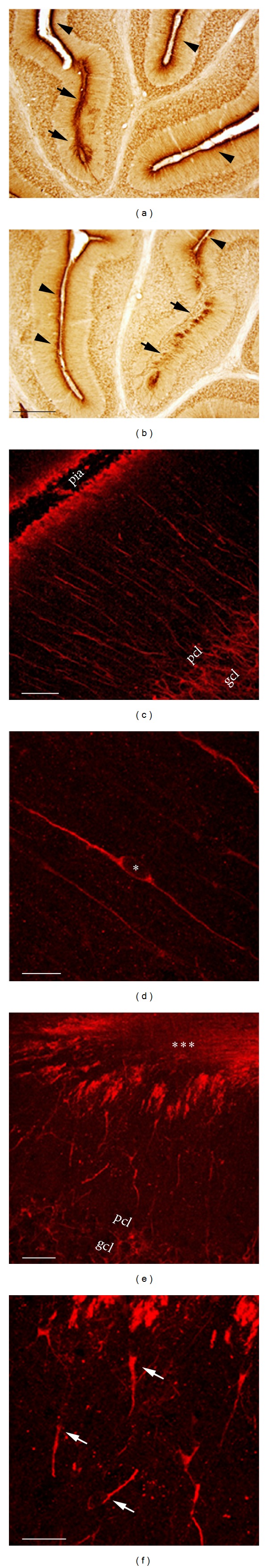
((a)-(b)) Altered expression pattern of Dcx in folia with heterotopia (arrows) compared to normal folia (arrowheads). ((c)-(d)) Higher magnification of Dcx expression in normal folia where migrating granule cells can be seen with bipolar morphologies. ((e)-(f)) Higher magnification of Dcx expression in heterotopia where migrating granule cells can be seen with abnormal morphologies. Asterisks denote areas lacking pia. Scale bars in microns: (a), (b) = 300; (c) = 37; (d) = 12; (e) = 32; (f) = 19.

Immunostaining of P14 tissue with heterotopia revealed abnormal distribution of doublecortin (Dcx) expression. As shown in Figure 2 (arrowheads in Figures 2(a), 2(b), and 2(c)), in unaffected folia Dcx was very dense along the pial surface and external granule cell layer (ECL). In addition, Dcx-expressing granule cells with bipolar morphologies could be observed in the molecular layer (Figure 2(d)). This morphology is indicative of migrating granule cells [19]. In contrast, MLH (arrows in Figures 2(a), 2(b), and 2(e)) were characterized by fusion of the pia and altered Dcx expression profiles. In particular, dense clusters of Dcx-expressing granule cells could be observed in the heterotopic molecular layer. Moreover, individual Dcx-expressing cells lacking the characteristic bipolar morphology were also evident near MLH. These data indicate that numerous Dcx-expressing granule cells have altered morphologies and fail to migrate out of the EGL. 

**Figure 3:**
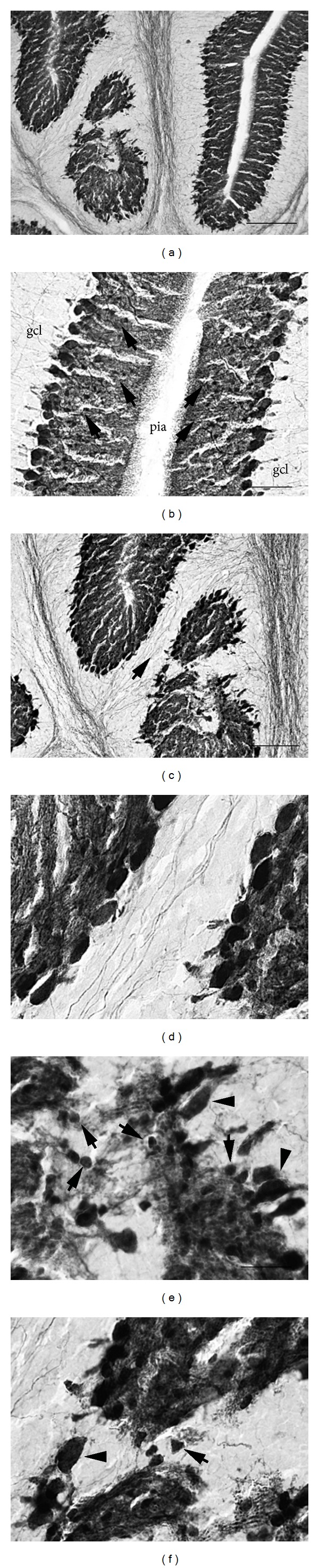
(a) Altered expression pattern of PV in folia with heterotopia (left side) compared to normal folia (right side). (b) Higher magnification of PV expression in normal folia where both Purkinje cells and molecular layer interneurons (arrows) are visible. ((c)–(f)) Higher magnification of PV expression in heterotopia where PV-labeled axons (arrow) are visible ((c), (d)) as well as molecular layer interneurons (arrows) and Purkinje cells (arrowheads). Scale bars in microns: (a) = 300; (b) = 75; (c) = 150; (d)–(f) = 125.

In addition to granule cells, previous reports have documented the presence of Purkinje cells, Golgi cells, and unipolar brush cells [11–13]. In order to determine whether additional cell types are present in heterotopia, we performed immunostaining for the calcium-binding protein parvalbumin (PV), as PV is expressed in Purkinje cells as well as molecular layer interneurons such as stellate cells and basket cells [20, 21]. As shown in Figure 3, heterotopia are indeed characterized by disorganization of PV-expressing Purkinje cells which were identified by their large somata (arrowheads in Figures 3(e) and 3(f)). PV-expressing axons from putative Purkinje cells were also observed in cases where heterotopia formed cellular bridges connecting adjacent granule cell layers in areas devoid of pia (Figures 3(c) and 3(d)). In addition, PV-expressing molecular layer interneurons with small somata were also present in heterotopia (arrows in Figures 3(e) and 3(f)). We also performed immunostaining for calretinin which labels Lugaro cells, unipolar brush cells, and granule cells [22–24]. We confirmed the presence of calretinin-expressing unipolar brush cells in MLH [12], but we found no presence of Lugaro cells in heterotopia (data not shown). Together with earlier studies, these data indicate that diverse neuronal classes are present in heterotopia including glutamate (such as granule cells and unipolar brush cells) and gamma aminobutyric acid-containing neurons (such as Golgi cells and molecular layer interneurons).

**Figure 4:**
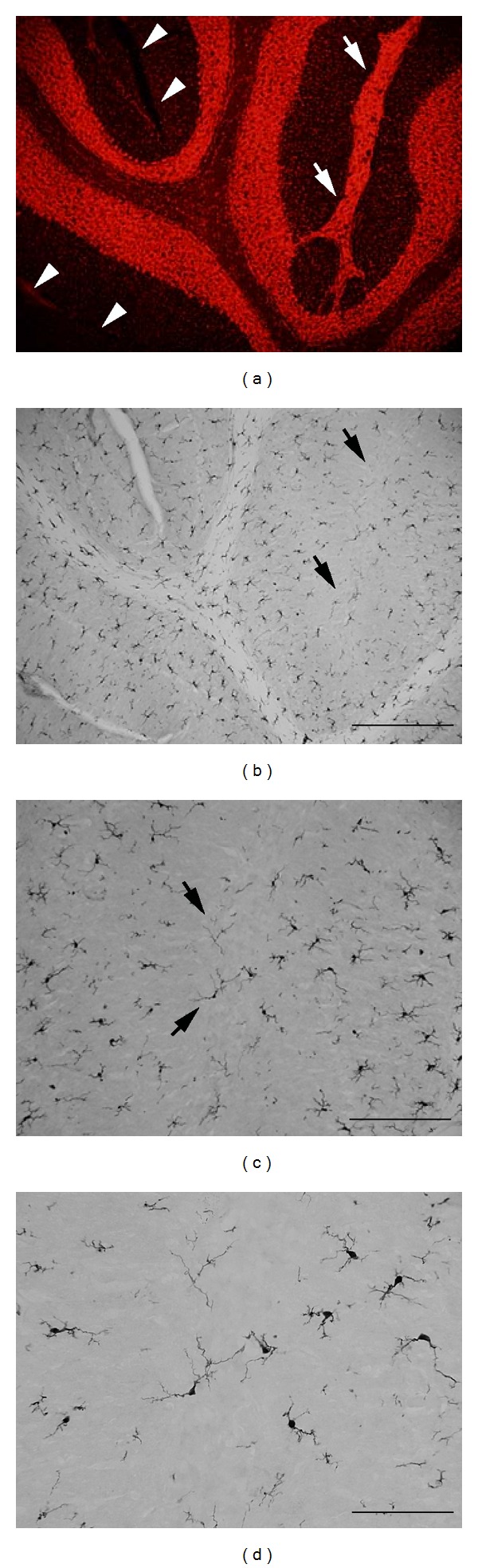
(a) Propidium iodide staining and Iba1 immunostaining ((b)–(d): same section as in (a)) demonstrate that microglia are present in heterotopia (arrows in (a), (b)). (c) Higher magnification of region in (b) indicated by arrows. (d) Higher magnification of region in (c) indicated by arrows. Scale bars in microns: (a), (b) = 200; (c) = 100; (d) = 50.

**Figure 5:**
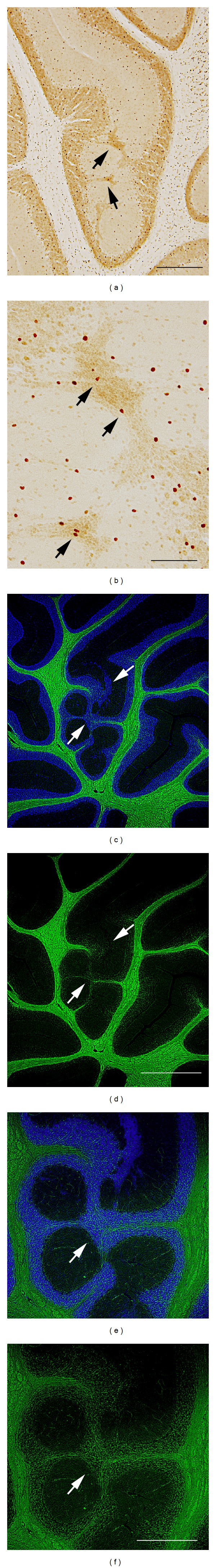
((a)-(b)) Olig2-expressing oligodendrocytes (arrow) are present in heterotopia. ((c), (e)) DAPI counterstaining and FluoroMyelin histochemistry ((d), (f): all same section) demonstrate that myelinated axons are present in heterotopia. ((e), (f)) Higher magnification of region in (d)-(e) indicated by arrows. Scale bars in microns: (a), (b) = 300; (c), (d) = 500.

 Three major glial cell types are present in the cerebellum including Bergmann glia, microglia, and oligodendrocytes. Bergmann glia expressing glial fibrillary acidic protein have been previously identified in MLH and were shown to have disorganized radial fibers [11] which may be responsible for the migration defect of granule cells. Consistent with this earlier finding, we found that the morphology of nestin-expressing radial glia is indeed altered in MLH (see Supplementary Figure 1 available in Supplementary material available online at http://dx.doi.org/10.1155/2013/805467). However, much less is known about the presence of other glial cell types in heterotopia. Therefore, we performed immunostaining for ionized calcium-binding adapter protein 1 (Iba1), which exclusively labels ramified microglia and is useful in determining *activation* of microglia via changes in staining intensity, soma size and shape, and ramification of processes [25, 26]. As shown in Figures 4(b)–4(d), microglia were also present in heterotopia but at surprisingly low levels compared to adjacent regions. In addition, labeled microglia in heterotopia did not appear to have activated morphologies with no obvious differences in morphology compared to microglia in adjacent unaffected folia. Immunostaining for oligodendrocyte transcription factor 2 (Olig2) was used to identify oligodendrocytes in MLH. As shown in Figures 5(a) and 5(b), Olig2-expressing oligodendrocytes were also present in MLH and were scattered among heterotopic granule cells. These data indicate that all three glial cell classes are found in MLH including Bergmann glia, oligodendrocytes, and microglia.

 In light of our observation of oligodendrocytes in MLH, we predicted that myelinated axons would be present in heterotopia. Therefore, fluorescent histochemistry for myelinated axons was performed. As shown in Figures 5(c) and 5(d), myelinated axons in unaffected folia are exclusively found in the granule cell layer and white matter. Not surprisingly, myelinated axons were also found in MLH. In cases where heterotopia formed a bridge of cells spanning the molecular layers of either side of primary fissure (between an area devoid of pia), we observed that myelinated axons also crossed these cell bridges (Figures 5(c)–5(f)). These data were also confirmed by immunostaining against myelin basic protein (data not shown).

**Figure 6:**
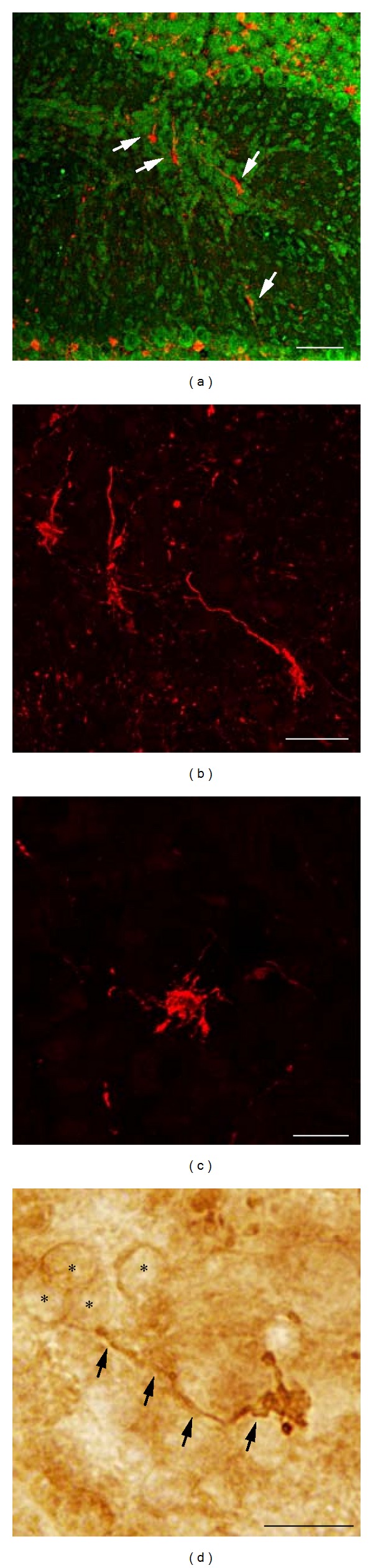
((a)-(b)): (same section) Calretinin-expressing axons (arrows) are present in heterotopia. Sytox green counterstaining shown in (b). ((c)-(d)) Higher magnification of calretinin axons and terminals. Asterisks in (d) denote weakly stained granule cells. Scale bars in microns: (a) = 52; (b) = 23.7; (c) = 12; (d) = 5.

As described above, myelinated fibers in unaffected folia were exclusively found in the granule cell layer indicating that these fibers arise from glutamatergic spinal and/or brainstem afferents and therefore constitute the mossy fiber projection [27]. Thus, the finding that myelinated fibers are present in MLH suggests that mossy fibers project onto heterotopic granule cells. Necchi and Scherini [12] described the presence of mossy fibers in heterotopia after phosphorylated neurofilament (200 kD) immunostaining, but Griffin et al. [10] did not observe mossy fibers following Golgi staining. In order to resolve these disparate results, we performed immunostaining for calretinin which labels mossy fiber axons and terminal rosettes [22, 23]. As shown in Figure 6, we did indeed identify calretinin-expressing axons with mossy fiber rosettes in heterotopia. These data indicate that heterotopic granule cells receive putative glutamatergic mossy fiber afferents from the spinal cord and/or brainstem as was suggested by Necchi and Scherini [12].

**Figure 7:**
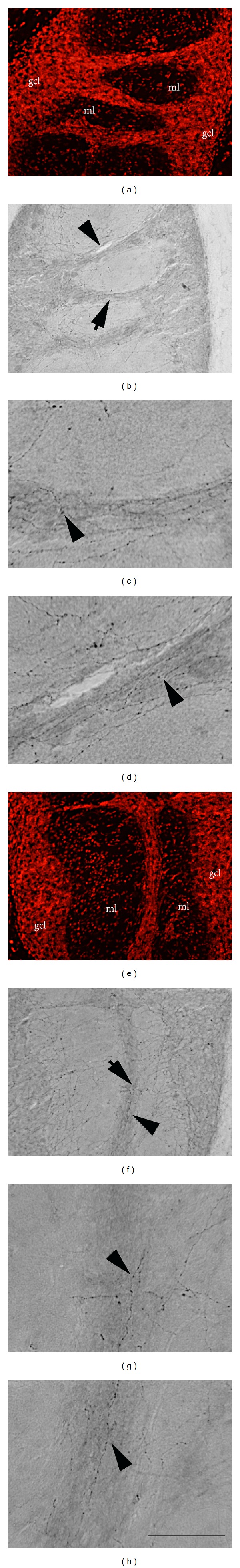
((a), (e)) Propidium iodide staining and immunostaining ((b)–(d): same section as in (a); (f)–(h): same section as in (e)) demonstrate that TH-expressing axons are present in heterotopia (arrow, arrowhead). (c) Higher magnification of section in (b) indicated by arrowhead. (d) Higher magnification of section in (b) indicated by arrow. Arrowheads in (c)-(d) point to axonal swellings. (g) Higher magnification of section in (f) indicated by arrow. (h) Higher magnification of section in (f) indicated by arrowhead. Arrowheads in (g)-(h) point to axonal swellings. Scale bars in microns: (a), (b), (e), and (f) = 300; (c), (d), (g), and (h) = 75.

A number of brainstem nuclei innervate the cerebellum and exert neuromodulatory influence over cerebellar function via catecholaminergic, serotonergic, and cholinergic signaling. We used immunocytochemistry to reveal tyrosine hydroxylase (TH) and serotonin transporter (5HTT) expression as a tool to label catecholaminergic and serotonergic axons in MLH. As shown in Figure 7, robust TH-labeled axons are indeed present in MLH. In cases where granule cells could be observed forming a bridge across a region devoid of pia (fused molecular layers), we observed TH-labeled axons that appeared to span this bridge of cells (Figures 7(a)–7(c)).

**Figure 8:**
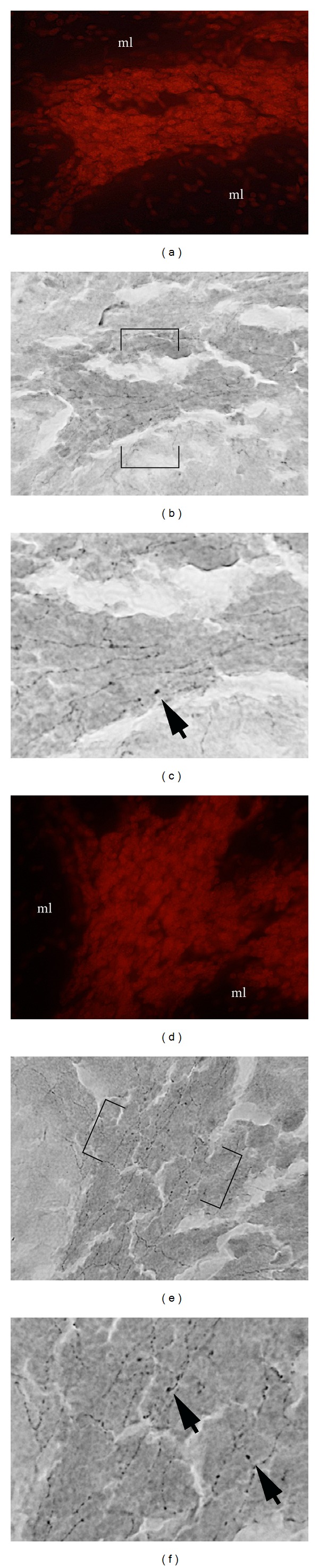
((a), (d)) Propidium iodide staining and immunostaining ((b)-(c): same section as in (a); (e)-(f): same section as in (d)) demonstrate that 5HTT-expressing axons are present in heterotopia (arrow). (c) Higher magnification of boxed section shown in (b) with arrows pointing to axonal swellings. Higher magnification of boxed section shown in (e) with arrows pointing to axonal swellings. Scale bars in microns: (a), (d) = 300; (b), (c), (e), and (f) = 50.

Compared to levels of TH expression, fewer and more fine-caliber 5HTT-labeled fibers were observed in granule cell layer of normal folia. However, as shown in Figures 8(c) and 8(f), 5HTT-labeled fibers were also present in MLH. In cases where granule cells could be observed forming a bridge between folia VIII and IX, we observed 5HTT-labeled axons that appeared to span this bridge of cells (data not shown). These data indicate that both catecholaminergic and serotonergic axons are present in MLH.

**Figure 9:**
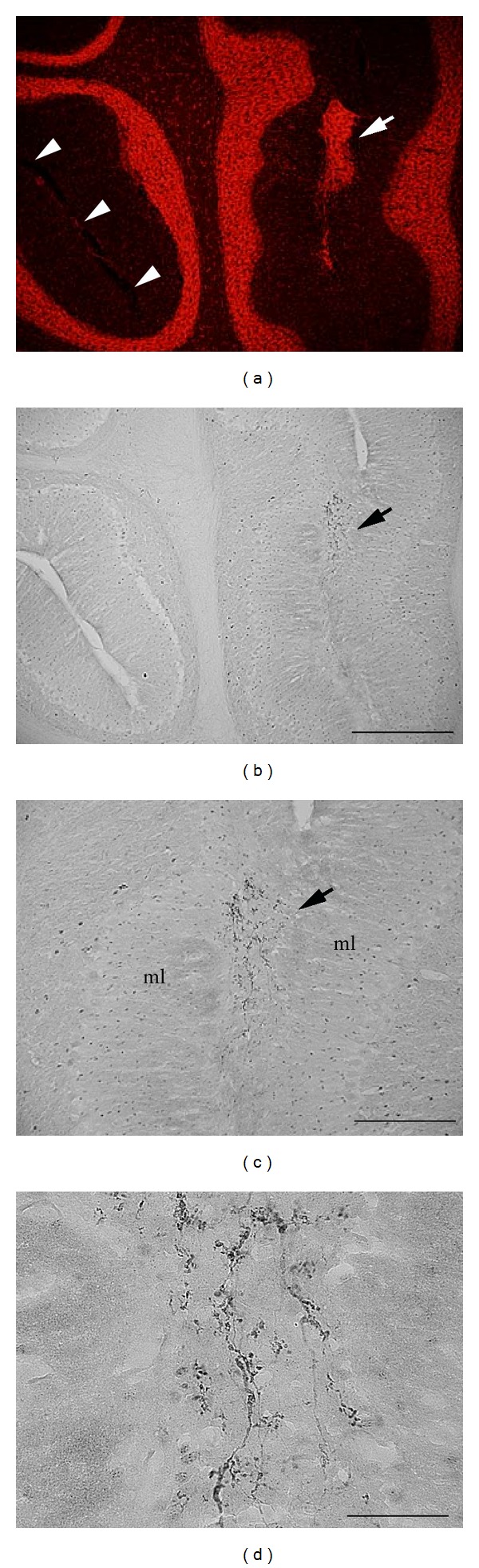
((a), (d)) Propidium iodide staining and immunostaining ((b)-(c): same section as in (a)) demonstrates that ChAT-expressing axons are present in heterotopia (arrow). ((b)–(d)) Higher magnification of section shown in (a) indicating that ChAT-expressing axons are embedded within heterotopic granule cells. Scale bars in microns: (a), (b) = 200; (c) = 100; (d) = 25.

During a literature search of the cholinergic innervation of the rat cerebellum, we identified two previous anecdotal sources of evidence that cholinergic axons are present in heterotopia. Firstly, Jaarsma and colleagues found that axons stained for choline acetyltransferase (ChAT) were present in MLH [28]. Secondly, plate 77 of the Paxinos and Watson rat brain atlas [29] has a photomicrograph of an acetylcholinesterase-stained sagittal section where labeled axons are clearly visible in MLH. As shown in Figure 9, we confirmed these observations with ChAT immunostaining, where labeled axons and terminals are clearly visible in heterotopia. Together with data described above, these findings indicate that diverse axons are present in heterotopia including myelinated, catecholaminergic, serotonergic, and cholinergic axons.

## 4. Discussion

 In the present report we provide further characterization of the cellular and axonal constituents of MLH of the rat cerebellar vermis. For example, we identified that in and surrounding heterotopia, Dcx-immunoreactive granule neurons in the molecular layer display abnormal morphology. In light of our observation of altered nestin-immunoreactive radial glial fibers, we propose the following model of cerebellar heterotopia formation in rat. First, for reasons yet unknown, small breaches of the leptomeninges develop in the cerebellar vermis along the base of primary fissure. Second, as a result of these breaches, radial glia morphology and the integrity of endfoot attachment forming the glial limitans are compromised. Finally, because radial glia provide the physical substrate for migrating granule cells, our model posits that defects in radial fibers then result in the failure of granule cells to exit the molecular layer/EGL. This model is supported by our observation of disorganized Dcx-expressing granule cells in heterotopia. These data also mimic our findings of MLH in the cerebellar vermis of mice [17, 18] which are also characterized by a lack of pial membrane, disrupted radial glia, and heterotopic granule cells in the molecular layer.

 Previous studies have identified several neuronal types in rat MLH including granule cells, Purkinje cells, Golgi cells, and unipolar brush cells [11–13]. In the present report we also identified PV-expressing molecular layer interneurons in MLH which likely include both basket cells and stellate cells. Therefore, our data suggest that molecular layer interneurons may provide inhibitory synaptic input to heterotopic granule cells which in the future should be verified with electrophysiological and/or electron micrographic methods. The effect of altered synaptic connections on the physiology of cerebellar neurons in heterotopia (as well as more broadly) remains unknown; however, these data demonstrate that there exist diverse neuronal groups in heterotopia. These data also point to similarities with MLH in mice which not only are comprised largely of granule cells but also contain Purkinje cells, Golgi cells, molecular layer interneurons, and unipolar brush cells [17].

 We show that nestin-expressing Bergmann glia and Olig2-expressing oligodendrocytes are present in MLH as well as Iba1-expressing microglia, indicating that three major classes of glial cells are present in heterotopia. These data are similar to the observations of heterotopia in mice [17]. Surprisingly, few microglia were found in heterotopia and those present therein did not have activated morphologies. These data suggest little, if any, immune or macrophagic activity of microglia in heterotopia [25, 26]. 

Consistent with the presence of oligodendrocytes in heterotopia, we observed myelinated axons in MLH. Myelinated axons in MLH may arise from glutamatergic spinal and/or brainstem cerebellar afferents, which is supported by our finding of calretinin-expressing rosettes in heterotopia. These findings are in contrast to the observations of Griffin et al. [10] who failed to find mossy fibers in heterotopia and who speculated that heterotopic granule cells do not receive mossy fiber projections [10]. However, in that study, Griffin et al. did not use immunocytochemistry for mossy fiber-specific markers.

Using immunocytochemistry for axon-specific markers, we show that diverse axons from brainstem neuromodulatory centers are found in MLH. For example, TH immunostaining revealed the presence of catecholaminergic axons in MLH, suggestive of dopaminergic and/or noradrenergic innervation. The likely sources of these neuromodulatory fibers include the ventral tegmental area [30] and locus coeruleus [31, 32], respectively. Likewise, 5HTT immunostaining revealed the presence of serotonergic fibers in MLH. The probable sources of these axons include the oral pontine and gigantocellular reticular nucleus [33]. Finally, we identified that ChAT- and acetylcholinesterase-expressing axons are present in heterotopia. Likely sources of these axons include the caudal medial vestibular nuclei and nucleus prepositus hypoglossus [34, 35]. Together, these data suggest diverse sources of neuromodulatory projections onto heterotopic granule cells from brainstem nuclei. Moreover, these data mirror the results from a recent study examining axonal classes in the mouse cerebellum, where we also observed TH-, 5HTT-, and ChAT-expressing axons in heterotopia [18]. Future studies should focus on detailing the synaptic connections onto heterotopic granule cells at the electron microscopic level. In addition, determining whether heterotopic granule cells extend appropriate parallel fibers and make synaptic connections onto the dendrites of Purkinje cells would provide additional clues as to how MLH affect cerebellar circuitry. 

Exactly how heterotopia affect cerebellar function and possibly manifest in behavioral changes of sensorimotor integration, postural control, motor learning, and so forth remains an open question. One prediction is that rats with heterotopia might be impaired on behavioral tests such as the balance beam task [36] or the ladder rung task [37]—a finding that would also shed new light on the importance of the PF on cerebellar control of sensorimotor function. Thus, MLH may affect interpretation of results of a wide range of behavioral studies where cerebellar function is involved in performance. For example, in an experiment where pharmacological agents are used to assess effects on motor behavior, MLH may increase the variability of behavioral scores leading to potential misinterpretation of the effects of the experimental intervention on behavior. Therefore, future studies should examine the role, if any, that cerebellar heterotopia of the PF has on behavior.

## Supplementary Material

Supplementary Figure 1: A-B (same section), Nestin labeled radial glia and vasculature in normal folia. C-D (same section), abnormally-organized Nestin labeled radial glia and vasculature in folia with heterotopia. Propidium iodide counterstaining shown in all left-side panels. Scalebars in microns: A-D = 75.
