# Supplementary figures and images for: Cellular and Axonal Diversity in Molecular Layer Heterotopia of the Rat Cerebellar Vermis
Source: Biomed Res Int. 2013 Sep 26;2013:805467. doi: 10.1155/2013/805467 (PMC3804155; doi:10.1155/2013/805467)

# Supplementary Figure 1

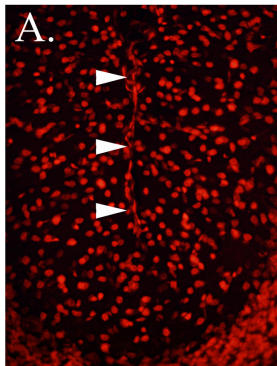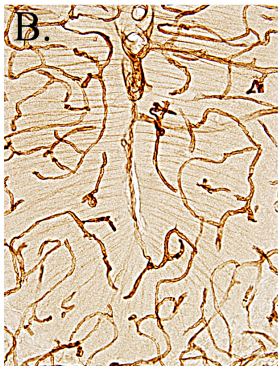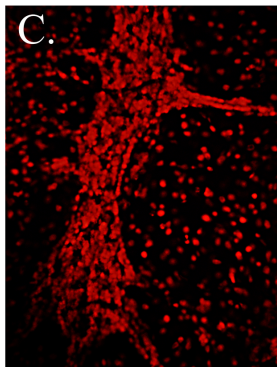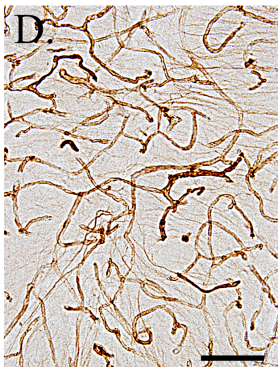

Supplement: Supplementary file 1 — Supplementary Figure 1: A-B (same section), Nestin labeled radial glia and vasculature in normal folia. C-D (same section), abnormally-organized Nestin labeled radial glia and vasculature in folia with heterotopia. Propidium iodide counterstaining shown in all left-side panels. Scalebars in microns: A-D = 75. [file 805467.f1.pdf]
